# Determinants of Intradialytic Phosphate Removal in Hemodiafiltration From Real‐World Clinical Practice

**DOI:** 10.1111/aor.70104

**Published:** 2026-02-02

**Authors:** David A. Jaques, Roohi Chhabra, Haalah Shaaker, Priyanka Khatri, Andrew Davenport

**Affiliations:** ^1^ Division of Nephrology and Hypertension Geneva University Hospitals Geneva Switzerland; ^2^ UCL Division of Medicine University College London London UK; ^3^ Clinical Nutrition Department King Abdulaziz University Jeddah Kingdom of Saudi Arabia; ^4^ Yong Loo Lin School of Medicine National University of Singapore Singapore Singapore; ^5^ Department of Medicine Alexandra Hospital Singapore Singapore; ^6^ UCL Centre for Kidney & Bladder Health, Royal Free Hospital University College London London UK

**Keywords:** clearance, convection, hemodiafiltration, hemodialysis, phosphate, removal

## Abstract

**Introduction:**

Hyperphosphatemia is frequent in hemodialysis (HD) patients and associates with cardiovascular mortality. Parameters of online hemodiafiltration (OL‐HDF) prescription influencing phosphate removal are poorly understood in real‐world clinical settings.

**Methods:**

We conducted a prospective observational study including chronic HD patients dialyzed at a single UK university hospital between December 2021 and July 2023. Phosphate removal was quantified from spent dialysate during a midweek session.

**Results:**

Dialysate phosphate was measured during 161 dialysis sessions in 128 unique patients (19 on HD and 109 on OL‐HDF), with mean age 63.3 ± 16.2 years and 77 (60.1%) men. Mean intra‐dialytic phosphate removal was 25.6 ± 14.3 mmol per session. In multivariate analysis, pre‐HD serum phosphate concentration, session time, and urea distribution volume (*V*) were positively associated with intradialytic phosphate removal. A significant interaction between session time and pre‐HD serum phosphate concentration indicated that longer sessions provided greater benefit to patients with higher baseline phosphate levels. Conversely, dialysis modality (OL‐HDF vs. HD) and substitution volume were not significantly associated with phosphate removal.

**Conclusions:**

OL‐HDF and substitution flow do not demonstrate a measurable beneficial impact on phosphate removal in a real‐world setting. Session time, pre‐HD serum phosphate concentration, and urea distribution volume (*V*) are the most relevant determinants of phosphate clearance in real‐world clinical practice. Session duration emerges as the most effective modifiable factor for controlling phosphate burden in dialysis patients, particularly for those with higher pre‐HD phosphate levels.

## Graphical abstract

We show that convective therapy in the form of hemodiafiltration does not demonstrate a measurable effect on phosphate removal in a real‐world setting. On the other hand, session duration is the most important factor in determining phosphate removal in hemodialysis patients.

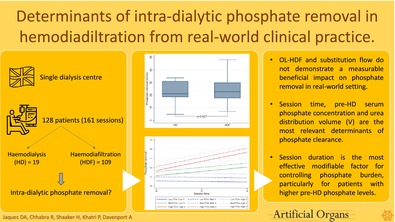

## 1 Introduction

Cardiovascular (CV) disease affects more than two‐thirds of end‐stage kidney disease (ESKD) patients receiving hemodialysis (HD) and remains the leading cause of mortality accounting for almost 50% of deaths in this population [1, 2]. Traditional risk factors alone do not fully explain the excess of CV mortality in ESKD patients [3]. Mineral bone disease (MBD) is common in this setting with evidence supporting a causal relationship with CV disease via the propensity for vascular calcification [4]. The *primum movens* of CKD‐MBD physiopathology is phosphate retention and higher phosphate concentrations are associated with increased mortality in European as well as North Americans ESKD dialysis patients [5, 6]. Limiting phosphate absorption is, however, difficult and mainly relies on restriction in dietary intake and use of oral binders. While intradialytic phosphate removal obviously plays a central role in phosphate homeostasis, this is usually not sufficient as most chronic HD patients are found to have serum phosphate levels above the normal range [7]. This indicates an unmet need to address this phosphate burden and its consequences in HD patients.

Phosphate removal during HD differs markedly from that of urea [8]. While serum urea concentration steadily decreases over the length of the dialysis session, phosphate levels tend to drop during the first hour of the session and then maintain a relatively stable concentration thereafter [9, 10]. This implies that, in contrast to urea, intradialytic phosphate removal is largely dependent on session time [11]. The impact of other dialysis parameters on phosphate removal is less well described. In particular, the added benefit of online hemodiafiltration (OL‐HDF) as compared to high‐flux HD on phosphate clearance is a matter of debate, with conflicting and inconclusive results from different studies [12]. Moreover, only a few small studies have considered phosphate removal based on measurement of spent dialysate [13, 14, 15, 16, 17, 18]. At the same time, a kinetic model predicted a 10% increase in phosphate removal with OL‐HDF as compared to HD [19]. As a result, clinicians are often faced with difficult decisions regarding the practical optimization of dialysis treatment to manage hyperphosphatemia in the era of OL‐HDF therapy.

Considering the importance of phosphate control in ESKD patients and the uncertainty regarding the interaction of various dialytic parameters on phosphate removal in real‐world practice, we conducted a prospective observational study to characterize the impact of convective therapy on phosphate clearance in dialysis patients attending for routine outpatient sessions.

## 2 Methods

### 2.1 Participants and Setting

We conducted a prospective observational study at a single dialysis center under the care of a university hospital in North London, UK. We screened all patients suffering from ESKD undergoing routine outpatient chronic dialysis treatments. Exclusion criteria were: (i) age < 18, (ii) cognitive impairment, (iii) unplanned hemodialysis initiation, and (iv) unable to provide informed consent. Patients were recruited between December 2021 and July 2023. For most patients, a single dialysis session per patient was considered. For some patients, however, more than one dialysis session was considered (see results).

### 2.2 Dialysis Prescription and Data Acquisition

Medical management and dialysis prescription were left to the discretion of attending physicians. Patients were not fasting. Samples were collected during a routine midweek dialysis session. Patients were dialyzed using Fresenius 5008H dialysis machines (Fresenius MC, Bad Homburg, Germany) with polysulfone high‐flux dialyzers (Fresenius MC, Bad Homburg, Germany) and ultrapure quality dialysis water. Dialysis sessions were tailored to achieve an equilibrated urea *Kt*/*V* of 1.4 when including residual kidney function. All patients were treated with a dialysate containing: acetate 3.0 mmol/L, bicarbonate 28 mmol/L, magnesium 0.5 mmol/L, and glucose 5.5 mmol/L. Dialysate calcium and potassium concentrations varied according to physician prescription. Potential dialysate calcium concentrations were 1.0, 1.25, and 1.75 mmol/L, while potential dialysate potassium concentrations were 1.0, 2.0, and 3.0 mmol/L. Dialysate sodium was again tailored according to physician prescription. No phosphate was contained in the dialysate. Dialysate temperature was set between 35° and 35.5°. Dialysate flow was set to 500 mL/min. When using OL‐HDF, post dilution reinjection was used while substitution fluid was generated online and added to total dialysate flow so that effective dialysate flow remained equal to 500 mL/min [20]. For HD, the total convection volume was equal to net ultrafiltration (UF) volume, whereas for OL‐HFD, the total convection volume was equal to the combined net UF volume and substitution volume.

A line was attached to the waste dialysate drain with continuous collection of the spent dialysate effluent in a container during the entire session. At the end of the session, the spent effluent was thoroughly mixed, and phosphate concentration measured. Intradialytic phosphate (PO_4_) removal in mmol was defined as phosphate outflow and calculated as follows:

$$\begin{array}{c} {\mathrm{PO}}_{4}\;\text{removal in}\;\mathrm{HD}\;\text{patients}={\mathrm{PO}}_{4}\;\text{effluent concentration}\\ \times \left(500\times \text{session time}+\mathrm{net}\;\mathrm{UF}\right)/1000 \end{array}$$

$$\begin{array}{c} {\mathrm{PO}}_{4}\;\text{removal in}\;\mathrm{HDF}\;\text{patients}={\mathrm{PO}}_{4}\;\text{effluent concentration}\\ \times \left(500\times \text{session time}+\text{substitution volume}+\mathrm{net}\;\mathrm{UF}\right)/1000 \end{array}$$

where PO_4_ effluent concentration is given in mmol/L, session time in min, substitution volume in mL, net UF in mL and 500 represents the total dialysate flow in mL/min. All laboratory analyses were conducted in a UK accredited laboratory (United Kingdom Accreditation Services (UKAS)). Phosphate was measured as phosphomolybdate by spectrophotometry using an ammonium molybdate method in an acidic buffer. The coefficient of variation for phosphate measurement at low concentrations is 6.3%. Urea clearance (*Kt*/*V*) was calculated according to the Daugirdas second generation equation [21]. Urea distribution volume (*V*) was estimated as 0.55 times body weight for males and 0.5 times body weight for females [22]. Finally, dialysis urea clearance (*K*) was derived from *Kt*/*V*, *V* and session time. Dialysis prescriptions and session details were gathered from the TMon electronic software (Fresenius MC, Bad Homburg, Germany).

### 2.3 Statistical Analysis

Continuous variables are expressed as mean ± standard deviation (SD) while categorical variables are expressed as number and relative frequencies. Baseline characteristics were compared between HD and OL‐HDF patients using Student's *t*‐test and chi‐squared for continuous and categorical variables, respectively. In regression models, intradialytic phosphate removal was considered as the dependent variable. Predictors of intradialytic potassium removal were characterized using univariate and multivariate linear regressions. Predictors were a priori specified based on prior scientific and theoretical knowledge as follows: Session time, pre‐HD serum phosphate concentration, net UF volume, dialyzer surface area, dialysis modality (HD vs. OL‐HDF), urea distribution volume (*V*), and dialysis urea clearance (*K*) [23]. In analyses restricted to OL‐HDF patients, substitution volume was considered instead of dialysis modality. As more than one dialysis session per patient was considered for some patients (see results), mixed‐effects regression models were used with a random intercept for patients to account for repeated measurements. Moreover, to account for a potential modification of effect, an interaction term between session time and pre‐HD serum phosphate concentration was introduced in regression models. Variables were transformed when necessary (log or square root). In regression models, continuous variables were standardized using a *z*‐score transform on a common scale with a mean of 0 and a SD of 1. Thus, *β* coefficients in regression models represent the expected change in the dependent variable per 1 SD change in the independent variable, allowing direct comparison of relative effect sizes [24]. Results are presented as *β* coefficient as well as associated 95% confidence intervals (CI) and *p* values. A two‐sided *p* value of < 0.05 was considered significant in every analysis. Patients with missing values on a priori selected predictors were excluded from the analyses, and no data were imputed. In linear regression models, homoscedasticity of residuals was assessed graphically with residual versus fitted plot. Collinearity was tested using variance inflation factors. Statistical analyses were conducted using Stata version 17 (StataCorp, College Station, TX, USA).

### 2.4 Ethics

This study was approved by a UK National Research Ethics Committee (21/NI/0059) and was carried out in accordance with the Declaration of Helsinki (2013), with all patients providing written informed consent. All patient data were coded prior to analysis.

## 3 Results

### 3.1 Baseline Characteristics

**FIGURE 1:**
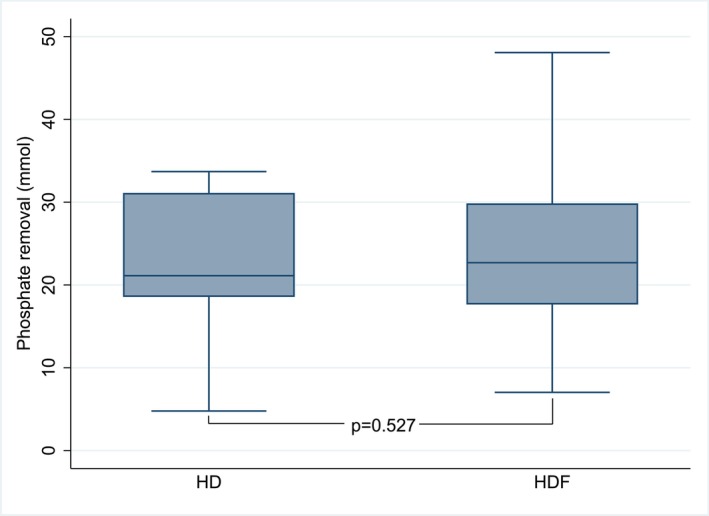
Intradialytic phosphate removal (mmol) according to dialysis modality (HD vs. OL‐HDF) (128 patients). HD, hemodialysis; OL‐HDF, online hemodiafiltration. [Color figure can be viewed at wileyonlinelibrary.com]

The study cohort consisted of 146 individual patients with phosphate removal measured during 187 dialysis sessions in total. Among those, 26 dialysis sessions had missing values on considered covariates. Excluding sessions with missing data, then 128 individual patients (19 HD and 109 OL‐HDF) with 161 dialysis sessions were included in the present study. Mean age of participants was 63.3 ± 16.2 years with 77 (60.1%) men. Mean sessional intradialytic phosphate removal was 25.6 ± 14.3 mmol. Patients' characteristics according to dialysis modality are presented in Table 1. Those characteristics were similar between groups except for substitution flow, substitution volume, total convection volume, and total effluent volume that were by definition higher with OL‐HDF as compared to HD patients. In particular, intradialytic phosphate removal was similar between patients treated with HD and OL‐HDF (Figure 1).

**TABLE 1:** Baseline characteristics according to dialysis modality (128 patients).

	HD (*N* = 19)	OL‐HDF (*N* = 109)	*p*
*Clinical*			
Age (years)	65.4 ± 15.5	62.9 ± 16.4	0.54
Gender (men)	11 (57.8%)	66 (60.5%)	0.82
Ethnicity			
Caucasian	8 (42.1%)	41 (37.6%)	0.56
Asian	9 (47.3%)	45 (41.2%)	
African	2 (10.5%)	23 (21.1%)	
Diabetes	6 (33.3%)	36 (37.5%)	0.73
Dry weight (kg)	72.0 ± 19.4	74.7 ± 18.0	0.56
BMI (kg/m^2^)	26.8 ± 6.4	26.6 ± 5.2	0.84
Phosphate binder	15 (78.9%)	82 (79.6%)	0.94
Vitamin D Substitute	17 (89.4%)	86 (79.6%)	0.31
Calcimimetic	3 (15.7%)	8 (7.4%)	0.23
*Laboratory*			
Hematocrit	0.35 ± 0.04	0.33 ± 0.03	0.12
C reactive protein (mg/L)	11.3 ± 13.5	10.0 ± 14.5	0.72
Pre‐HD bicarbonate (mmol/L)	21.7 ± 2.5	20.8 ± 2.7	0.21
Pre‐HD calcium (mmol/L)	2.18 ± 0.20	2.16 ± 0.17	0.75
Pre‐HD phosphate (mmol/L)	1.74 ± 0.56	1.79 ± 0.55	0.66
Serum albumin (g/L)	40.3 ± 4.1	37.9 ± 4.9	0.05
Parathormone (pmol/L)	31 (15–47)	36 (15–60)	0.57
*Dialysis*			
Vintage (months)	49 (24–82)	20 (8–55)	**0.01**
Session time (min)	209 ± 38	209 ± 27	0.94
Blood flow (mL/min)	288 ± 34	289 ± 31	0.94
Substitution flow (mL/min)	0	82.7 ± 25.9	**0.00**
Substitution volume (L)	0	17.2 ± 6.0	**0.00**
Net UF volume (mL)	1489 ± 709	1552 ± 995	0.79
Total convective volume (L)	1.4 ± 0.7	18.8 ± 6.4	**0.00**
Total effluent volume (L)	106.2 ± 19.4	123.3 ± 17.0	**0.00**
Dialyzer surface (m^2^)	2.08 ± 0.35	1.95 ± 0.30	0.07
Urea *Kt*/*V*	1.31 ± 0.38	1.36 ± 0.35	0.56
nPCR (g/kg/day)	0.92 ± 0.80	0.98 ± 0.28	0.42
Effluent phosphate (mmol/L)	0.22 ± 0.09	0.20 ± 0.10	0.59
Intra‐HD phosphate removal (mmol)	23.7 ± 11.2	25.9 ± 14.8	0.52

*Note:* Bold values indicate *p* < 0.05.

Abbreviations: BMI, body mass index; HD, hemofiltration; nPCR, normalized protein catabolic rate; OL‐HDF, online hemodiafiltration; UF, ultrafiltration.

### 3.2 Intra‐Dialytic Phosphate Removal: Impact of Dialysis Modality

We first analyzed the determinants of intradialytic phosphate removal in the whole study cohort (128 patients and 161 sessions) (Table 2).

**TABLE 2:** Determinants of intradialytic phosphate removal (mmol) in univariate and multivariate analysis (128 patients and 161 sessions).

	Univariate model			Multivariate model		
	*β* coefficient	95% CI	*p*	*β* coefficient	95% CI	*p*
Pre‐HD phosphate	0.52	0.38 to 0.65	**0.00**	0.53	0.41 to 0.65	**0.00**
Session time (*t*)	0.26	0.11 to 0.42	**0.00**	0.23	0.08 to 0.37	**0.00**
Session time × pre‐HD phosphate	0.16	0.04 to 0.28	**0.00**	0.13	0.03 to 0.22	**0.00**
*V*	0.27	0.12 to 0.42	**0.00**	0.21	0.07 to 0.35	**0.00**
Net UF volume	0.18	0.02 to 0.34	**0.02**	0.08	−0.05 to 0.22	0.22
Dialyzer surface	0.20	0.04 to 0.35	**0.01**	0.02	−0.12 to 0.16	0.75
OL‐HDF (vs. HD)	0.27	−0.16 to 0.71	0.22	0.07	−0.26 to 0.41	0.67
*K*	0.05	−0.10 to 0.22	0.48	−0.08	−0.22 to 0.05	0.24

*Note:* Variables are all standardized to a mean of 0 and a standard deviation of 1 to allow direct comparison of effect sizes. Bold values indicate *p* < 0.05.

Abbreviations: HD, hemofiltration; *K*, dialysis urea clearance; OL‐HDF, online hemodiafiltration; UF, ultrafiltration; *V*, urea distribution volume.

In univariate analysis, the following variables were positively associated with intra‐dialytic phosphate removal: Pre‐HD serum phosphate concentration, session time, urea distribution volume (*V*), net UF volume, and dialyzer surface area. Moreover, the interaction of session time with pre‐HD serum phosphate concentration was significant, indicating that the effect of pre‐HD serum phosphate concentration on intradialytic phosphate removal was dependent on session time. Finally, dialysis urea clearance (*K*) and dialysis modality (OL‐HDF vs. HD) were not associated with intradialytic phosphate removal.

**FIGURE 2:**
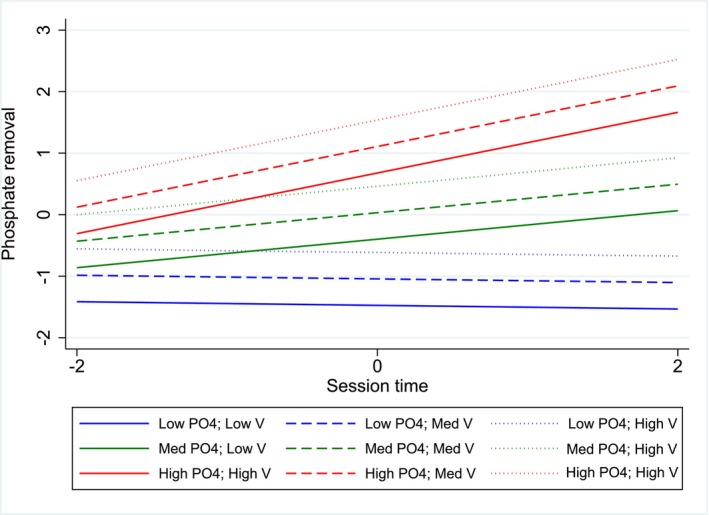
Multivariate association between intradialytic phosphate removal and session time (*t*), pre‐HD serum phosphate concentration as well as urea distribution volume (*V*) (128 patients and 161 sessions). Variables are all standardized to a mean of 0 and a standard deviation of 1 to allow direct comparison of effect sizes. In this setting, “low” designates −2 of standardized distribution, “med” designates 0 of standardized distribution, and “high” designates 2 of standardized distribution. Model is adjusted for the following covariates: Net UF volume, dialyzer surface, dialysis modality (OL‐HDF vs. HD), dialysis urea clearance (*K*), and urea distribution volume (*V*). HD, hemodialysis; OL‐HDF, online hemodiafiltration; PO_4_, phosphate; UF, ultrafiltration; *V*, urea distribution volume. [Color figure can be viewed at wileyonlinelibrary.com]

In multivariate analysis, the following variables were positively associated with intradialytic phosphate removal (in decreasing order of effect size): Pre‐HD serum phosphate concentration, session time, and urea distribution volume (*V*). Moreover, the interaction of session time with pre‐HD serum phosphate concentration was significant, indicating that the effect of pre‐HD serum phosphate concentration on intradialytic phosphate removal was dependent on session time. Finally, dialysis urea clearance (*K*), net UF volume, dialyzer surface area, and dialysis modality (OL‐HDF vs. HF) were not associated with intra‐dialytic phosphate removal. Variance inflation factors analysis showed no collinearity in the final multivariate model. Homoscedasticity was satisfied in the final multivariate model. The association between intradialytic phosphate removal and session time, pre‐HD serum phosphate concentration, as well as urea distribution volume in the final multivariate model is illustrated in Figure 2.

These data indicate that dialysis modality (OL‐HDF as opposed to HD) is not associated with intradialytic phosphate removal.

### 3.3 Intra‐Dialytic Phosphate Removal: Impact of Substitution Flow

We next analyzed the determinants of intradialytic phosphate removal in the subgroup of patients on OL‐HDF treatment (109 patients and 134 sessions) (Table 3).

**TABLE 3:** Determinants of intradialytic phosphate removal (mmol) in OL‐HDF patients in univariate and multivariate analysis (109 patients and 134 sessions).

	Univariate model			Multivariate model		
	*β* coefficient	95% CI	*p*	*β* coefficient	95% CI	*p*
Pre‐HD phosphate	0.55	0.41 to 0.68	**0.00**	0.51	0.38 to 0.65	**0.00**
Session time (*t*)	0.25	0.07 to 0.42	**0.00**	0.18	0.01 to 0.36	**0.03**
Session time × pre‐HD phosphate	0.28	0.11 to 0.46	**0.00**	0.14	−0.00 to 0.29	0.05
*V*	0.18	0.01 to 0.35	**0.02**	0.18	0.02 to 0.34	**0.02**
Net UF volume	0.14	−0.02 to 0.30	0.08	0.05	−0.09 to 0.20	0.49
Dialyzer surface	0.18	0.01 to 0.34	**0.03**	−0.02	−0.19 to 0.15	0.81
Substitution volume	0.23	0.00 to 0.46	**0.04**	0.12	−0.09 to 0.33	0.27
*K*	0.01	−0.15 to 0.18	0.83	−0.07	−0.22 to 0.08	0.36

*Note:* Variables are all standardized to a mean of 0 and a standard deviation of 1 to allow direct comparison of effect sizes. Bold values indicate *p* < 0.05.

Abbreviations: HD, hemofiltration; *K*, dialysis urea clearance; OL‐HDF, online hemodiafiltration; UF, ultrafiltration; *V*, urea distribution volume.

In univariate analysis, the following variables were positively associated with intradialytic phosphate removal: Pre‐HD serum phosphate concentration, session time, urea distribution volume (*V*), dialyzer surface area, and substitution volume. Moreover, the interaction of session time with pre‐HD serum phosphate concentration was significant, indicating that the effect of pre‐HD serum phosphate concentration on intradialytic phosphate removal was dependent on session time. Finally, net UF volume and dialysis urea clearance (*K*) were not associated with intradialytic phosphate removal.

**FIGURE 3:**
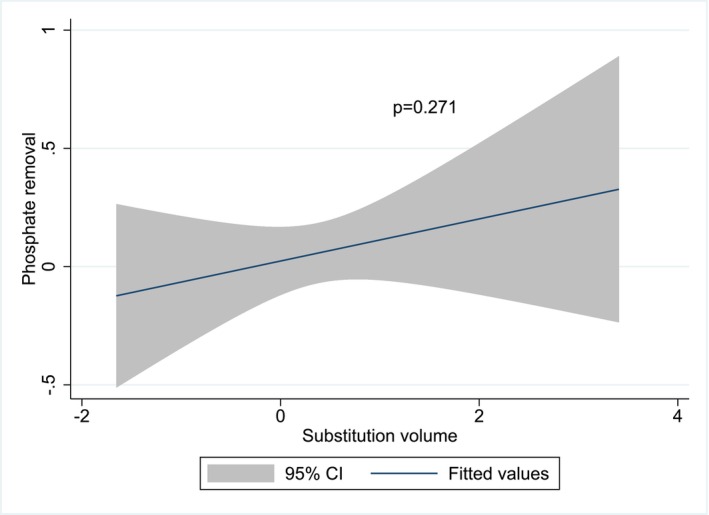
Multivariate association between intradialytic phosphate removal and volume of substitution in OL‐HDF patients (109 patients and 134 sessions). Variables are all standardized to a mean of 0 and a standard deviation of 1 to allow direct comparison of effect sizes. Model is adjusted for the following covariates: Session time, pre‐HD serum phosphate concentration, net UF volume, dialyzer surface, dialysis urea clearance (*K*) and urea distribution volume (*V*). HD, hemodialysis; OL‐HDF, online hemodiafiltration; PO_4_, phosphate; UF, ultrafiltration; *V*, urea distribution volume. [Color figure can be viewed at wileyonlinelibrary.com]

In multivariate analysis, the following variables were positively associated with intra‐dialytic phosphate removal (in decreasing order of effect size): Pre‐HD serum phosphate concentration, session time, and urea distribution volume (*V*). Dialysis urea clearance (*K*), net UF volume, dialyzer surface area, and substitution volume were not associated with intra‐dialytic phosphate removal. Variance inflation factors analysis showed no collinearity in the final multivariate model. Homoscedasticity was satisfied in the final multivariate model. The association between intradialytic phosphate removal and volume of substitution in OL‐HDF patients in the final multivariate model is illustrated in Figure 3.

These data indicate that the volume of substitution is not associated with intradialytic phosphate removal in OL‐HDF patients.

### 3.4 Sensitivity Analysis

We considered the association between hematocrit and intra‐dialytic phosphate removal in a sensitivity analysis. In univariate analysis, hematocrit was negatively associated with intradialytic phosphate removal (Table S1). When added to our multivariate model, hematocrit maintained a negative association with intradialytic phosphate removal. Associations between other variables and intradialytic phosphate removal were not qualitatively modified when hematocrit was added to the multivariate model (Table S1).

## 4 Discussion

In this study, we characterized the determinants of intra‐dialytic phosphate removal in OL‐HDF therapy based on data from real world clinical practice. Pre‐HD serum phosphate concentration, session time and urea distribution volume are the most relevant determinants of phosphate clearance in this setting. On the opposite, OL‐HDF and substitution flow do not have a significant impact on phosphate removal. These findings should guide clinicians in their routine care of dialysis patients.

Dietary restrictions are considered pivotal to phosphate management in ESKD dialysis patients [25]. However, limiting phosphate intake could lead to avoidance of otherwise healthy food as well as excessive restriction of dietary protein intake [26, 27]. Not surprisingly, this also significantly impacts on patient health related quality of life [28]. Although phosphate binders can potentially reduce serum phosphate concentration in dialysis patients, their prescription significantly increases pill burden and frequently induces gastrointestinal side effects [29]. This explains the very low adherence to these medications in the dialysis population [30]. Consequently, it is observed that most ESKD dialysis patients have serum phosphate concentrations above target range [7]. In this setting, it is of prime importance for the clinician to identify the specific dialysis parameters affecting phosphate removal in daily practice. Session time has been recognized as an important predictor of intradialytic phosphate removal, but other parameters are less well described [11]. Moreover, recent advances in OL‐HDF techniques have added an extra layer of complexity to this issue [19, 31]. Thus, data from the real world on the interplay between determinants of intradialytic phosphate removal during OL‐HDF therapy are lacking.

In the present study, we measured intradialytic phosphate removal based on spent dialysate analysis in established ESKD dialysis patients regularly treated with high‐flux HD or OL‐HDF therapy. Overall, pre‐HD serum phosphate concentration was the single most important factor associated with intradialytic phosphate removal. This finding aligns with the fact that the theoretical removal of any solute during dialysis is limited by its concentration in the inflowing blood. Thus, a higher initial serum phosphate concentration would allow a larger contribution of this phenomenon to the total intradialytic removal. A similar phenomenon has been well described for other small solutes including potassium [32].

Session time was the second most important factor associated with phosphate clearance. As dialysis is started, serum phosphate concentration is expected to rapidly decrease for the first 60 to 90 min, but level off thereafter to a relatively steady concentration for the remainder of the session [33]. This later phase is characterized by an equilibrium between dialytic removal and recruitment of the phosphate pool from intracellular space and deeper compartments [33]. Thus, contrary to other solutes such as urea, as serum phosphate levels are relatively maintained during dialysis, total phosphate removal is highly dependent on session time [34, 35]. While our findings confirmed this theoretical model, we also importantly observed a significant interaction between session time and pre‐HD serum phosphate concentration. In other words, the effect of session time on phosphate removal was not uniform, but depended on the patient's baseline phosphate level, highlighting that patients with higher pre‐HD phosphate derived greater benefit from longer sessions. On the other hand, session time was not associated with intradialytic phosphate removal in patients with lower pre‐HD serum phosphate levels. While we could not find a prior description of this phenomenon in the related literature, our results are in agreement with the theory of phosphate homeostasis in dialysis patients. Specifically, the rate limiting step of phosphate clearance in the later part of a dialysis session is the transfer from the intracellular to the extracellular space [33]. Conceptually, a higher pre‐HD serum phosphate concentration would reflect a larger overall phosphate content, with the initial drop in concentration allowing for later mobilization of deeper compartments throughout the dialysis session.

For a thorough analysis, we separated total dialysis urea clearance (*Kt*/*V*) in its distinct components. The influence of session time (*t*) was discussed above while dialytic urea clearance by itself (*K*) did not significantly influence phosphate removal. Urea distribution volume (*V*), on the other hand, strongly associated with phosphate removal in our cohort. Here again, these findings are in agreement with theoretical intra‐dialytic phosphate kinetic, with compartments transfer being the rate‐limiting step, and not the phosphate clearance of the dialyzer itself [33]. As a larger urea distribution volume (*V*) associated with greater intradialytic phosphate removal, it can be postulated that patients with larger body mass would maintain a higher phosphate transfer from intracellular to extracellular spaces during dialysis, thereby allowing for mobilization of deeper compartments [11]. In this setting, it is pivotal to highlight that dialysis urea clearance, as routinely measured by *Kt*/*V*, could be largely dissociated from actual intradialytic removal of small solutes and thus misleading in assessing overall dialysis efficiency [11]. This phenomenon has been described regarding middle molecules (beta2‐microglobulin), but also small molecules including phosphate, potassium, urea, and creatinine [11, 32]. Overall, it can be concluded based on our results that longer sessions, as well as larger urea distribution volume (*V*), allow greater shift of phosphate out of the extraplasmatic compartments, allowing a higher absolute solute removal that would not be adequately captured by measurement of urea *Kt*/*V*. Given those complex dynamics during dialysis, accurate measurement of solute removal requires the analysis of spent dialysate collection.

The added benefit of OL‐HDF prescription on intra‐dialytic phosphate removal is debated. In some studies, OL‐HDF was associated with lower pre‐HD serum phosphate levels as compared to HD [36, 37, 38]. In others, no difference was shown [39, 40, 41]. However, pre‐HD serum solute levels can be influenced by diet, residual kidney function, and various drugs. Consequently, accurate quantification of solute removal should rely on the analysis of spent dialysate, and only a limited number of small‐sized observational studies have done so [13, 14, 15, 16, 17, 18]. In most of those studies, a slight increase in phosphate removal was observed with OL‐HDF as compared to HD [14, 15, 16, 17]. However, some of these studies used low‐flux hemodialysis or high‐flux dialysis but with low beta2‐microglobulin clearances. A single small study of 12 patients surprisingly reported an increased phosphate removal of 44% with OL‐HDF while a substitution flow of only 50 mL/min was prescribed [18]. Using a dedicated kinetic model, Daugirdas recently predicted a 9% increase in intra‐dialytic phosphate removal using 100 mL/min post‐dilution OL‐HDF as compared to HD [19]. We could not confirm those results in our cohort. On the opposite, we observed that prescription of OL‐HDF as well as the importance of post‐dilution substitution volume did not have a significant measurable impact on phosphate removal in our patients. Differences are unlikely to be attributed to insufficient convection as mean substitution flow was 83 mL/min in our OL‐HDF patients. Rather, we believe that in a real‐world clinical practice, the impact of convective therapy on phosphate removal is negligible as compared to the very strong determinants of phosphate clearance, which are session duration and total body phosphate content (as represented by pre‐HD serum phosphate concentration and urea distribution volume (*V*)). We do not refute a possible theoretical advantage of HDF on phosphate removal. However, we believe that convective therapy should not be prescribed for the sole purpose of improving phosphate control, and clinicians should rather focus on dialytic parameters with clear and impactful effects, with session time as the main modifiable factor.

All studies have potential limitations, and most patients in our study had intradialytic phosphate removal measured during a single session. A higher proportion of repeated measurements per patient could have better controlled for intraindividual variability. Second, urea distribution volume (*V*) was not directly measured but rather estimated by a simple anthropomorphic formula. However, similar methodology is used in comparable studies and reflects real‐world practice. Moreover, it is highly unlikely that a somewhat more precise estimation of urea distribution volume (*V*) would have altered the highly significant association reported in this study. Third, treatment was individually tailored accounting for potential residual kidney function. This may explain the slightly shorter session times and lower convection volumes as compared to a standard prescription of a 4‐h session. Theoretically, our findings might not directly apply to longer, more intensive sessions, although qualitative discrepancies with our results in this setting seem unlikely. Fourth, the single‐center nature of our study does not allow direct generalization of our results to other settings. Finally, precise modeling of peri‐dialytic phosphate kinetics would have required phosphate values at multiple timepoints that were not available in this study. Such mechanistic considerations were however not the purpose of the present study, as we focused on the clinical determinants of phosphate removal during dialysis in a real‐world setting.

## 5 Conclusion

In conclusion, in this prospective observational study, we analyzed the determinants of dialysis prescription in regard to phosphate removal in a real‐world clinical setting comparing patients treated with OL‐HDF and high‐flux HD. We observed that convective therapy in the form of OL‐HDF was not significantly associated with improved phosphate clearance in established ESKD dialysis patients. On the contrary, session duration was a major contributor to phosphate clearance along with pre‐HD serum phosphate concentration and urea distribution volume (*V*) as proxies of total phosphate content. Moreover, the influence of session time itself depended on pre‐HD serum levels, with longer sessions mainly benefitting patients with higher pre‐HD phosphate levels. Clinicians should bear those findings in mind when tailoring prescription to their individual patients, as longer sessions should be regarded as the most efficient measure to control phosphate burden in a real‐world clinical practice.

## Author Contributions

D.A.J. analyzed the data, interpreted the results, and wrote the manuscript. R.C., H.S., and P.K. recruited patients and collected the data. A.D. designed the study, interpreted the results, and revised the manuscript.

## Funding

The authors have nothing to report.

## Conflicts of Interest

The authors declare no conflicts of interest.

## Supporting information


**Table S1:** Determinants of intradialytic phosphate removal (mmol) in univariate and multivariate analysis.

## Data Availability

The data that support the findings of this study are available from the corresponding author upon reasonable request.

## References

[1] R. Boenink , V. S. Stel , B. E. Waldum‐Grevbo , et al., “Data From the ERA‐EDTA Registry Were Examined for Trends in Excess Mortality in European Adults on Kidney Replacement Therapy,” Kidney International 98, no. 4 (2020): 999–1008. 32569654 10.1016/j.kint.2020.05.039

[2] S. Thompson , M. James , N. Wiebe , et al., “Cause of Death in Patients With Reduced Kidney Function,” Journal of the American Society of Nephrology 26, no. 10 (2015): 2504–2511. 25733525 10.1681/ASN.2014070714 PMC4587695

[3] S. Said and G. T. Hernandez , “The Link Between Chronic Kidney Disease and Cardiovascular Disease,” Journal of Nephropathology 3, no. 3 (2014): 99–104. 25093157 10.12860/jnp.2014.19 PMC4119330

[4] K. C. Reimer , J. Nadal , H. Meiselbach , et al., “Association of Mineral and Bone Biomarkers With Adverse Cardiovascular Outcomes and Mortality in the German Chronic Kidney Disease (GCKD) Cohort,” Bone Research 11, no. 1 (2023): 1–8. 37857629 10.1038/s41413-023-00291-8 PMC10587182

[5] M. B. Lopes , A. Karaboyas , B. Bieber , et al., “Impact of Longer Term Phosphorus Control on Cardiovascular Mortality in Hemodialysis Patients Using an Area Under the Curve Approach: Results From the DOPPS,” Nephrology, Dialysis, Transplantation: Official Publication of the European Dialysis and Transplant Association—European Renal Association 35, no. 10 (2020): 1794–1801. 32594171 10.1093/ndt/gfaa054 PMC7538234

[6] J. Floege , J. Kim , E. Ireland , et al., “Serum iPTH, Calcium and Phosphate, and the Risk of Mortality in a European Haemodialysis Population,” Nephrology, Dialysis, Transplantation: Official Publication of the European Dialysis and Transplant Association—European Renal Association 26, no. 6 (2011): 1948–1955. 20466670 10.1093/ndt/gfq219 PMC3107766

[7] M. Guedes , B. Bieber , I. Dasgupta , et al., “Serum Phosphorus Level Rises in US Hemodialysis Patients Over the Past Decade: A DOPPS Special Report,” Kidney Medicine 5, no. 2 (2023): 100584. 36704450 10.1016/j.xkme.2022.100584 PMC9871331

[8] J. P. Gutzwiller , D. Schneditz , A. R. Huber , C. Schindler , F. Gutzwiller , and C. E. Zehnder , “Estimating Phosphate Removal in Haemodialysis: An Additional Tool to Quantify Dialysis Dose,” Nephrology, Dialysis, Transplantation: Official Publication of the European Dialysis and Transplant Association—European Renal Association 17, no. 6 (2002): 1037–1044. 12032194 10.1093/ndt/17.6.1037

[9] C. A. DeSoi and J. G. Umans , “Phosphate Kinetics During High‐Flux Hemodialysis,” Journal of the American Society of Nephrology 4, no. 5 (1993): 1214–1218. 8305649 10.1681/ASN.V451214

[10] H. Pogglitsch , W. Petek , E. Ziak , F. Sterz , and H. Holzer , “Phosphorus Kinetics During Haemodialysis and Haemofiltration,” Proceedings of the European Dialysis and Transplant Association ‐ European Renal Association 21 (1985): 461–468. 3991541

[11] S. Eloot , W. Van Biesen , A. Dhondt , et al., “Impact of Hemodialysis Duration on the Removal of Uremic Retention Solutes,” Kidney International 73, no. 6 (2008): 765–770. 18160958 10.1038/sj.ki.5002750

[12] F. Locatelli , F. Carfagna , L. Del Vecchio , and V. La Milia , “Haemodialysis or Haemodiafiltration: That Is the Question,” Nephrology, Dialysis, Transplantation: Official Publication of the European Dialysis and Transplant Association—European Renal Association 33, no. 11 (2018): 1896–1904. 29688552 10.1093/ndt/gfy035

[13] W. Zhang , Q. Du , J. Xiao , et al., “Modification and Validation of the Phosphate Removal Model: A Multicenter Study,” Kidney & Blood Pressure Research 46, no. 1 (2021): 53–62. 33477164 10.1159/000511375

[14] F. Švára , F. Lopot , I. Valkovský , and O. Pecha , “Phosphorus Removal in Low‐Flux Hemodialysis, High‐Flux Hemodialysis, and Hemodiafiltration,” ASAIO Journal 62, no. 2 (2016): 176–181. 26579979 10.1097/MAT.0000000000000313

[15] T. Cornelis , F. M. van der Sande , S. Eloot , et al., “Acute Hemodynamic Response and Uremic Toxin Removal in Conventional and Extended Hemodialysis and Hemodiafiltration: A Randomized Crossover Study,” American Journal of Kidney Diseases: The Official Journal of the National Kidney Foundation 64, no. 2 (2014): 247–256. 24698199 10.1053/j.ajkd.2014.02.016

[16] Y. Nagaoka , H. Matsumoto , T. Okada , et al., “Benefits of First‐Half Intensive Haemodiafiltration for the Removal of Uraemic Solutes,” Nephrology (Carlton, Vic.) 16, no. 5 (2011): 476–482. 21126287 10.1111/j.1440-1797.2010.01431.x

[17] W. Lornoy , J. De Meester , I. Becaus , J. M. Billiouw , P. A. Van Malderen , and M. Van Pottelberge , “Impact of Convective Flow on Phosphorus Removal in Maintenance Hemodialysis Patients,” Journal of Renal Nutrition: Official Journal of the Council on Renal Nutrition of the National Kidney Foundation 16, no. 1 (2006): 47–53. 10.1053/j.jrn.2005.10.008 16414441

[18] R. Minutolo , V. Bellizzi , M. Cioffi , et al., “Postdialytic Rebound of Serum Phosphorus: Pathogenetic and Clinical Insights,” Journal of the American Society of Nephrology: JASN 13, no. 4 (2002): 1046–1054. 11912265 10.1681/ASN.V1341046

[19] J. T. Daugirdas , “Comparison of Measured vs Kinetic‐Model Predicted Phosphate Removal During Hemodialysis and Hemodiafiltration,” Nephrology, Dialysis, Transplantation: Official Publication of the European Dialysis and Transplant Association—European Renal Association 37, no. 12 (2022): 2522–2527. 35869975 10.1093/ndt/gfac223

[20] F. Maduell , R. Ojeda , M. Arias‐Guillén , et al., “Optimization of Dialysate Flow in On‐Line Hemodiafiltration,” Nefrologia: Publicacion Oficial de la Sociedad Espanola de Nefrologia 35, no. 5 (2015): 473–478. 10.1016/j.nefro.2015.06.019 26306957

[21] J. T. Daugirdas , “Second Generation Logarithmic Estimates of Single‐Pool Variable Volume Kt/V: An Analysis of Error,” Journal of the American Society of Nephrology: JASN 4, no. 5 (1993): 1205–1213. 8305648 10.1681/ASN.V451205

[22] F. Maduell , F. Sigüenza , A. Caridad , F. Miralles , and F. Serrato , “Analysis of Urea Distribution Volume in Hemodialysis,” Nephron 66, no. 3 (1994): 312–316. 8190184 10.1159/000187829

[23] J. T. Daugirdas , “Removal of Phosphorus by Hemodialysis,” Seminars in Dialysis 28, no. 6 (2015): 620–623. 26358370 10.1111/sdi.12439

[24] J. Cohen , P. Cohen , S. G. West , and L. S. Aiken , Applied Multiple Regression/Correlation Analysis for the Behavioral Sciences, 3rd ed. (Routledge, 2013) 536 p.

[25] K. Kalantar‐Zadeh , D. Forfang , G. Bakris , K. J. Martin , S. M. Moe , and S. M. Sprague , “Managing Phosphate Burden in Patients Receiving Dialysis: Beyond Phosphate Binders and Diet,” Kidney360 4, no. 11 (2023): 1650–1656. 37870525 10.34067/KID.0000000000000262 PMC10695651

[26] M. Rufino , E. de Bonis , M. Martín , et al., “Is It Possible to Control Hyperphosphataemia With Diet, Without Inducing Protein Malnutrition?,” Nephrology, Dialysis, Transplantation 13, no. Suppl 3 (1998): 65–67. 10.1093/ndt/13.suppl_3.65 9568824

[27] G. Khoueiry , A. Waked , M. Goldman , et al., “Dietary Intake in Hemodialysis Patients Does Not Reflect a Heart Healthy Diet,” Journal of Renal Nutrition 21, no. 6 (2011): 438–447. 21185740 10.1053/j.jrn.2010.09.001

[28] K. Woolley , A. Fishbach , and R. M. Wang , “Food Restriction and the Experience of Social Isolation,” Journal of Personality and Social Psychology 119, no. 3 (2020): 657–671. 31724417 10.1037/pspi0000223

[29] S. Wang , E. A. Anum , K. Ramakrishnan , T. Alfieri , P. Braunhofer , and B. Newsome , “Reasons for Phosphate Binder Discontinuation Vary by Binder Type,” Journal of Renal Nutrition 24, no. 2 (2014): 105–109. 24462496 10.1053/j.jrn.2013.11.004

[30] Y. W. Chiu , I. Teitelbaum , M. Misra , E. M. de Leon , T. Adzize , and R. Mehrotra , “Pill Burden, Adherence, Hyperphosphatemia, and Quality of Life in Maintenance Dialysis Patients,” Clinical Journal of the American Society of Nephrology 4, no. 6 (2009): 1089–1096. 19423571 10.2215/CJN.00290109 PMC2689877

[31] P. J. Blankestijn , R. W. M. Vernooij , C. Hockham , et al., “Effect of Hemodiafiltration or Hemodialysis on Mortality in Kidney Failure,” New England Journal of Medicine 389, no. 8 (2023): 700–709. 37326323 10.1056/NEJMoa2304820

[32] D. A. Jaques , R. Chhabra , P. Khatri , and A. Davenport , “Impact of Convective Clearance on Intra‐Dialytic Potassium Removal in Chronic Dialysis Patients,” Artificial Organs 49, no. 2 (2025): 300–309. 39377155 10.1111/aor.14883 PMC11752973

[33] R. Pohlmeier and J. Vienken , “Phosphate Removal and Hemodialysis Conditions,” Kidney International. Supplement 78 (2001): S190–S194. 11169009 10.1046/j.1523-1755.2001.59780190.x

[34] R. Elias , V. R. C. Alvares , and R. Moysés , “Phosphate Removal During Conventional Hemodialysis: A Decades‐Old Misconception,” Kidney & Blood Pressure Research 43 (2018): 110–114. 29414834 10.1159/000487108

[35] M. S. Sampaio , F. Ruzany , D. M. Dorigo , and J. H. R. Suassuna , “Phosphate Mass Removal During Hemodialysis: A Comparison Between eKT/V‐Matched Conventional and Extended Dialysis,” American Journal of Nephrology 36, no. 2 (2012): 121–126. 22776782 10.1159/000338675

[36] H. Schiffl , “Prospective Randomized Cross‐Over Long‐Term Comparison of Online Haemodiafiltration and Ultrapure High‐Flux Haemodialysis,” European Journal of Medical Research 12, no. 1 (2007): 26–33. 17363355

[37] M. P. C. Grooteman , M. A. van den Dorpel , M. L. Bots , et al., “Effect of Online Hemodiafiltration on All‐Cause Mortality and Cardiovascular Outcomes,” Journal of the American Society of Nephrology: JASN 23, no. 6 (2012): 1087–1096. 22539829 10.1681/ASN.2011121140 PMC3358764

[38] M. Morena , A. Jaussent , L. Chalabi , et al., “Treatment Tolerance and Patient‐Reported Outcomes Favor Online Hemodiafiltration Compared to High‐Flux Hemodialysis in the Elderly,” Kidney International 91, no. 6 (2017): 1495–1509. 28318624 10.1016/j.kint.2017.01.013

[39] V. Wizemann , C. Lotz , F. Techert , and S. Uthoff , “On‐Line Haemodiafiltration Versus Low‐Flux Haemodialysis. A Prospective Randomized Study,” Nephrology, Dialysis, Transplantation: Official Publication of the European Dialysis and Transplant Association—European Renal Association 15, no. Suppl 1 (2000): 43–48. 10.1093/oxfordjournals.ndt.a027963 10737166

[40] E. Ok , G. Asci , H. Toz , et al., “Mortality and Cardiovascular Events in Online Haemodiafiltration (OL‐HDF) Compared With High‐Flux Dialysis: Results From the Turkish OL‐HDF Study,” Nephrology, Dialysis, Transplantation: Official Publication of the European Dialysis and Transplant Association—European Renal Association 28, no. 1 (2013): 192–202. 23229932 10.1093/ndt/gfs407

[41] F. Maduell , F. Moreso , M. Pons , et al., “High‐Efficiency Postdilution Online Hemodiafiltration Reduces All‐Cause Mortality in Hemodialysis Patients,” Journal of the American Society of Nephrology: JASN 24, no. 3 (2013): 487–497. 23411788 10.1681/ASN.2012080875 PMC3582206

