# Supplementary material for: Determinants of Intradialytic Phosphate Removal in Hemodiafiltration From Real‐World Clinical Practice
Source: Artif Organs. 2026 Feb 2;50(5):755–63. doi: 10.1111/aor.70104 (PMC13206526; doi:10.1111/aor.70104)
Supplement: Supplementary file 1 — Table S1: Determinants of intradialytic phosphate removal (mmol) in univariate and multivariate analysis. [file AOR-50-755-s001.docx]

**Supplementary Table S1** : Determinants of intra-dialytic phosphate removal (mmol) in univariate and multivariate analysis.

|  | **Univariate model** | | | **Multivariate model** | | |
| --- | --- | --- | --- | --- | --- | --- |
|  | **β coefficient** | **95% CI** | **P value** | **β coefficient** | **95% CI** | **P value** |
| Pre-HD phosphate | 0.52 | 0.38 to 0.65 | **0.00** | 0.53 | 0.41 to 0.65 | **0.00** |
| Session time (t) | 0.26 | 0.11 to 0.42 | **0.00** | 0.23 | 0.08 to 0.37 | **0.00** |
| Session time X pre-HD phosphate | 0.16 | 0.04 to 0.28 | **0.00** | 0.13 | 0.03 to 0.22 | **0.00** |
| V | 0.27 | 0.12 to 0.42 | **0.00** | 0.21 | 0.07 to 0.35 | **0.00** |
| Net UF volume | 0.18 | 0.02 to 0.34 | **0.02** | 0.08 | -0.05 to 0.22 | 0.22 |
| Dialyzer surface | 0.20 | 0.04 to 0.35 | **0.01** | 0.02 | -0.12 to 0.16 | 0.75 |
| OL-HDF (vs HD) | 0.27 | -0.16 to 0.71 | 0.22 | 0.07 | -0.26 to 0.41 | 0.67 |
| K | 0.05 | -0.10 to 0.22 | 0.48 | -0.08 | -0.22 to 0.05 | 0.24 |
| Hematocrit | -0.20 | -0.36 to -0.05 | **0.00** | -0.17 | -0.29 to -0.05 | **0.00** |

Variables are all standardized to a mean of 0 and a standard deviation of 1 to allow direct comparison of effect sizes.

Bold values indicate p<0.05.

*Abbreviations: HD, hemofiltration; OL-HDF, online hemodiafiltration ; K, dialysis urea clearance; UF, ultrafiltration; V,* urea distribution volume*.*
